# *trans*-Dimethano­lbis(1,1,1-trifluoro-5,5-dimethyl­hexane-2,4-dionato)zinc(II)

**DOI:** 10.1107/S1600536808037963

**Published:** 2008-12-10

**Authors:** Gerald O. Hunter, Matthias Zeller, Brian D. Leskiw

**Affiliations:** a Youngstown State University, Department of Chemistry, 1 University Plaza, Youngstown, OH 44555, USA

## Abstract

The title compound, [Zn(C_8_H_10_F_3_O_2_)_2_(CH_4_O)_2_], is a dimethanol coordinated zinc complex with the acetyl acetonate derivative 1,1,1-trifluoro-5,5-dimethyl­hexane-2,4-dionate. The *bis­*-β-diketonate complex, which is isostructural with its Co analogue, is located on a crystallographic inversion center. The complex is octa­hedral with basically no distortion, and the methanol mol­ecules are in *trans* positions with respect to one another. The planes of the β-diketonate and the ZnO_4_ unit are tilted by 18.64 (10)° against each other. O—H⋯O hydrogen bonds between the methanol hydroxyl groups and neighboring diketonate O atoms create chains running along [100].

## Related literature

For information regarding the synthesis of various metal β-diketonates refer to Watson & Lin (1966 ▶). For mass spectrometry related articles see Lerach & Leskiw (2008 ▶) and Schildcrout (1976 ▶). For a variety of applications and properties of metal β-diketonate complexes refer to Burtoloso (2005 ▶), Katok *et al.* (2006 ▶) and Condorelli *et al.* (2007 ▶). Lerach *et al.* (2007 ▶) report the structure of the Co analogue of the title compound.
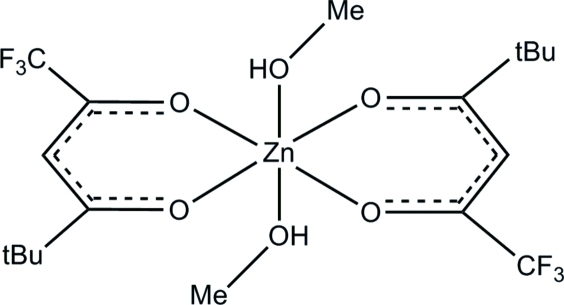


## Experimental

### 

#### Crystal data

[Zn(C_8_H_10_F_3_O_2_)_2_(CH_4_O)_2_]
*M* *_r_* = 519.79
Triclinic, 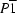

*a* = 5.470 (2) Å
*b* = 8.755 (3) Å
*c* = 12.031 (4) Å
α = 78.785 (5)°
β = 80.542 (5)°
γ = 88.083 (5)°
*V* = 557.5 (3) Å^3^
*Z* = 1
Mo *K*α radiation
μ = 1.18 mm^−1^
*T* = 100 (2) K
0.55 × 0.26 × 0.05 mm

#### Data collection

Bruker SMART APEX CCD diffractometer
Absorption correction: multi-scan (*APEX2*; Bruker, 2008 ▶) *T*_min_ = 0.603, *T*_max_ = 0.943
5584 measured reflections
2736 independent reflections
2103 reflections with *I* > 2σ(*I*)
*R*_int_ = 0.044

#### Refinement

*R*[*F* ^2^ > 2σ(*F* ^2^)] = 0.052
*wR*(*F* ^2^) = 0.114
*S* = 1.04
2736 reflections
149 parameters
1 restraint
H atoms treated by a mixture of independent and constrained refinement
Δρ_max_ = 0.85 e Å^−3^
Δρ_min_ = −0.97 e Å^−3^

### 

Data collection: *APEX2* (Bruker, 2008 ▶); cell refinement: *APEX2*; data reduction: *APEX2*; program(s) used to solve structure: *SHELXTL* (Sheldrick, 2008 ▶); program(s) used to refine structure: *SHELXTL*; molecular graphics: *SHELXTL*; software used to prepare material for publication: *SHELXTL*.

## Supplementary Material

Crystal structure: contains datablocks I, global. DOI: 10.1107/S1600536808037963/lx2078sup1.cif

Structure factors: contains datablocks I. DOI: 10.1107/S1600536808037963/lx2078Isup2.hkl

Additional supplementary materials:  crystallographic information; 3D view; checkCIF report

## Figures and Tables

**Table 1:** Hydrogen-bond geometry (Å, °)

*D*—H⋯*A*	*D*—H	H⋯*A*	*D*⋯*A*	*D*—H⋯*A*
O3—H3*A*⋯O1^i^	0.82 (2)	2.06 (2)	2.869 (3)	168 (4)

Symmetry code: (i) 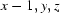.

**Fig. 1.**
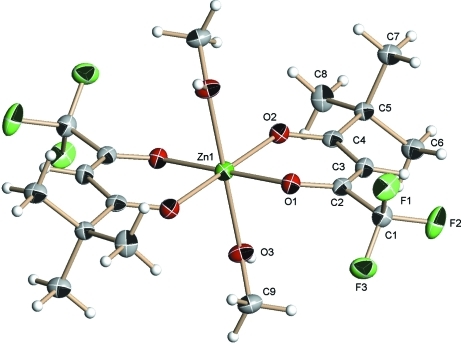
ORTEP representation of the asymmetric unit of the title compound (50% probability displacement ellipsoids). H atoms are shown as circles of arbitrary radii.

## supplementary crystallographic information

### Comment

β-Diketonates and especially metal β-diketonate complexes have been widely studied for both their instrinsic properties as well as a variety of scientific and technolgical applications. Especially interesting applications include, but are not limited to, catalysis (Burtoloso, 2005), carbon-nanotube structures (Katok *et al.*, 2006), or the deposition of metallic or ceramic thin films (Condorelli *et al.*, 2007). In our own laboratory we are investigating gas phase reactions of a series of metal acetylacetonate (acac) complexes. Through mass spectrometric analysis, several acetylacetonate and substituted acetyl acetonate species were observed to undergo various reactions including ligand exchange and association (Schildcrout, 1976; Lerach & Leskiw, 2008). In this context fluorinated metal-β-diketonates are especially interesting because of their increased volality, thermal stability, and also their ease of preparation.

The title compound, [Zn(C_8_H_10_F_3_O_2_)_2_(CH_3_OH)_2_], which is isostructural with its Co analogue (Lerach *et al.*, 2007) is a dimethanol coordinate of a zinc complex with the ligand 1,1,1-trifluoro-5,5-dimethylhexane-2,4-dionate, an acetyl acetonate derivative with each a *tert*-butyl and a trifluoromethyl substituent. A thermal ellipsoid plot of the molecule is shown in Fig. 1. The bis-β-diketonate complex is located on a crystallographic inversion center with the two methanol molecules in *trans* position to each other. The coordination environment of the central zinc cation is octahedral with only a very slight distortion: angles around the Zn atom deviate from 90° by 0.36 (8)° or less, and Zn—O distances are 2.054 (2) and 2.040 (2) Å for the zinc β-diketonate bonds and 2.161 (2) Å towards the methanol molecules. The mean planes of the diketonate ligands, defined by the atoms O1, O2 and C1 to C5, and that of the ZnO_4_ unit are tilted against each other by an angle of 18.64 (10)°, which is virtually identical to the vaule of 17.41 (7)° observed in the structure of the Co analogue of the title compound.

Packing of the molecules within the structure is assisted by hydrogen bonds between the methanol hydroxyl groups and diketonate oxygen atoms of neighboring molecules (Table 1). The O—H···O interactions create hydrogen bonded chains that stretch along the *a*-axis of the structure.

### Experimental

The synthesis of the title compound was adapted from Watson & Lin (1966). 0.80 ml (5.0 mmol) of the ligand were added to a stirring solution of 0.22 g of ZnCl_2_ (1.6 m mol) and 50 ml of de-inoized water. Diluted 1:1 (*v*/*v*) NH_4_OH was added dropwise to the mixture until no more visible precipitate formed. The solution was stirred for another hour at room temperature, and the precipitate was isolated by vacuum filtration. The desired product was re-crystallized by overnight evaporation of a concentrated methanolic solution.

### Refinement

The hydroxyl H atom was located in a difference density Fourier map and the O—H distance was restrained to 0.84 (2) Å. All other H atoms were placed in calculated positions with C—H distances of 0.98 (methyl) and 0.95 Å (CH). The methyl and hydroxyl H's were refined with an isotropic displacement parameter *U*_iso_ of 1.5 times *U*_eq_ of the adjacent carbon or oxygen atom, and the C—H hydrogen atom with *U*_iso_ = 1.2 *U*_eq_(C). Methyl hydrogen atoms were allowed to rotate to best fit the experimental electron density.

### Crystal data

**Table:**

[Zn(C_8_H_10_F_3_O_2_)_2_(CH_4_O)_2_]	*Z* = 1
*M**_r_* = 519.79	*F*(000) = 268
Triclinic, *P*1	*D*_x_ = 1.548 Mg m^−^^3^
Hall symbol: -P 1	Mo *K*α radiation, λ = 0.71073 Å
*a* = 5.470 (2) Å	Cell parameters from 1117 reflections
*b* = 8.755 (3) Å	θ = 2.4–29.6°
*c* = 12.031 (4) Å	µ = 1.18 mm^−^^1^
α = 78.785 (5)°	*T* = 100 K
β = 80.542 (5)°	Plate, colourless
γ = 88.083 (5)°	0.55 × 0.26 × 0.05 mm
*V* = 557.5 (3) Å^3^	

### Data collection

**Table:**

Bruker SMART APEX CCD diffractometer	2736 independent reflections
Radiation source: fine-focus sealed tube	2103 reflections with *I* > 2σ(*I*)
graphite	*R*_int_ = 0.044
Detector resolution: 10.0 pixels mm^-1^	θ_max_ = 28.3°, θ_min_ = 1.8°
ω scans	*h* = −7→7
Absorption correction: multi-scan (*APEX2*; Bruker, 2008)	*k* = −11→11
*T*_min_ = 0.603, *T*_max_ = 0.943	*l* = −15→16
5584 measured reflections	

### Refinement

**Table:**

Refinement on *F*^2^	Primary atom site location: structure-invariant direct methods
Least-squares matrix: full	Secondary atom site location: difference Fourier map
*R*[*F*^2^ > 2σ(*F*^2^)] = 0.052	Hydrogen site location: difference Fourier map
*wR*(*F*^2^) = 0.114	H atoms treated by a mixture of independent and constrained refinement
*S* = 1.04	*w* = 1/[σ^2^(*F*_o_^2^) + (0.0494*P*)^2^ + 0.1141*P*] where *P* = (*F*_o_^2^ + 2*F*_c_^2^)/3
2736 reflections	(Δ/σ)_max_ < 0.001
149 parameters	Δρ_max_ = 0.85 e Å^−^^3^
1 restraint	Δρ_min_ = −0.97 e Å^−^^3^

### Special details

**Table:**

Geometry. All e.s.d.'s (except the e.s.d. in the dihedral angle between two l.s. planes) are estimated using the full covariance matrix. The cell e.s.d.'s are taken into account individually in the estimation of e.s.d.'s in distances, angles and torsion angles; correlations between e.s.d.'s in cell parameters are only used when they are defined by crystal symmetry. An approximate (isotropic) treatment of cell e.s.d.'s is used for estimating e.s.d.'s involving l.s. planes.
Refinement. Refinement of *F*^2^ against ALL reflections. The weighted *R*-factor *wR* and goodness of fit *S* are based on *F*^2^, conventional *R*-factors *R* are based on *F*, with *F* set to zero for negative *F*^2^. The threshold expression of *F*^2^ > σ(*F*^2^) is used only for calculating *R*-factors(gt) *etc*. and is not relevant to the choice of reflections for refinement. *R*-factors based on *F*^2^ are statistically about twice as large as those based on *F*, and *R*- factors based on ALL data will be even larger.

### Fractional atomic coordinates and isotropic or equivalent isotropic displacement parameters (Å^2^)

**Table:**

	*x*	*y*	*z*	*U*_iso_*/*U*_eq_	
C1	1.1698 (6)	1.2211 (4)	0.7642 (3)	0.0245 (7)	
C2	1.0749 (5)	1.0950 (3)	0.7105 (3)	0.0196 (6)	
C3	0.9063 (5)	0.9904 (3)	0.7791 (3)	0.0211 (6)	
H3	0.8553	1.0023	0.8563	0.025*	
C4	0.8021 (5)	0.8648 (3)	0.7425 (3)	0.0199 (6)	
C5	0.6476 (5)	0.7405 (3)	0.8316 (3)	0.0200 (6)	
C6	0.4765 (6)	0.8131 (4)	0.9221 (3)	0.0266 (7)	
H6A	0.3670	0.8895	0.8840	0.040*	
H6B	0.5768	0.8647	0.9643	0.040*	
H6C	0.3764	0.7312	0.9758	0.040*	
C7	0.8326 (6)	0.6306 (4)	0.8926 (3)	0.0265 (7)	
H7A	0.7410	0.5512	0.9521	0.040*	
H7B	0.9354	0.6910	0.9278	0.040*	
H7C	0.9389	0.5801	0.8364	0.040*	
C8	0.4976 (6)	0.6471 (4)	0.7721 (3)	0.0298 (8)	
H8A	0.3999	0.5681	0.8297	0.045*	
H8B	0.6103	0.5961	0.7184	0.045*	
H8C	0.3861	0.7171	0.7300	0.045*	
C9	0.7190 (6)	1.3221 (4)	0.5071 (3)	0.0290 (7)	
H9A	0.7125	1.3480	0.5832	0.044*	
H9B	0.5854	1.3768	0.4702	0.044*	
H9C	0.8793	1.3542	0.4599	0.044*	
F1	1.4150 (3)	1.2087 (2)	0.76434 (17)	0.0350 (5)	
F2	1.0662 (4)	1.2202 (2)	0.87310 (16)	0.0385 (5)	
F3	1.1312 (4)	1.3633 (2)	0.70461 (18)	0.0384 (5)	
O1	1.1718 (4)	1.1047 (2)	0.60505 (17)	0.0214 (5)	
O2	0.8347 (4)	0.8468 (2)	0.64018 (18)	0.0223 (5)	
O3	0.6892 (4)	1.1570 (2)	0.51870 (19)	0.0252 (5)	
H3A	0.547 (4)	1.129 (4)	0.548 (3)	0.038*	
Zn1	1.0000	1.0000	0.5000	0.01973 (16)	

### Atomic displacement parameters (Å^2^)

**Table:**

	*U*^11^	*U*^22^	*U*^33^	*U*^12^	*U*^13^	*U*^23^
C1	0.0202 (15)	0.0285 (17)	0.0260 (17)	−0.0026 (13)	−0.0032 (13)	−0.0083 (13)
C2	0.0129 (13)	0.0233 (15)	0.0249 (16)	0.0025 (11)	−0.0068 (11)	−0.0075 (12)
C3	0.0172 (14)	0.0256 (16)	0.0212 (15)	0.0002 (12)	−0.0031 (12)	−0.0067 (12)
C4	0.0109 (13)	0.0223 (15)	0.0257 (16)	0.0043 (11)	−0.0019 (11)	−0.0040 (12)
C5	0.0163 (14)	0.0205 (15)	0.0227 (16)	−0.0004 (11)	−0.0018 (12)	−0.0039 (12)
C6	0.0196 (15)	0.0272 (16)	0.0301 (17)	0.0004 (13)	0.0005 (13)	−0.0024 (14)
C7	0.0196 (15)	0.0253 (16)	0.0323 (18)	−0.0009 (12)	−0.0015 (13)	−0.0020 (14)
C8	0.0251 (16)	0.0338 (18)	0.0301 (18)	−0.0146 (14)	−0.0014 (14)	−0.0048 (14)
C9	0.0271 (17)	0.0235 (16)	0.0370 (19)	0.0037 (13)	−0.0037 (14)	−0.0088 (14)
F1	0.0192 (9)	0.0440 (12)	0.0478 (13)	−0.0032 (8)	−0.0100 (9)	−0.0191 (10)
F2	0.0398 (12)	0.0472 (13)	0.0315 (11)	−0.0171 (10)	0.0070 (9)	−0.0227 (10)
F3	0.0513 (13)	0.0216 (10)	0.0473 (13)	0.0004 (9)	−0.0196 (10)	−0.0093 (9)
O1	0.0172 (10)	0.0255 (11)	0.0222 (11)	−0.0015 (8)	−0.0026 (8)	−0.0067 (9)
O2	0.0211 (10)	0.0221 (11)	0.0241 (11)	−0.0026 (8)	−0.0028 (9)	−0.0059 (9)
O3	0.0148 (10)	0.0226 (11)	0.0374 (13)	0.0007 (9)	0.0000 (9)	−0.0076 (10)
Zn1	0.0150 (3)	0.0215 (3)	0.0228 (3)	−0.00167 (19)	−0.00203 (19)	−0.0050 (2)

### Geometric parameters (Å, °)

**Table:**

C1—F2	1.338 (4)	C7—H7B	0.9800
C1—F3	1.339 (4)	C7—H7C	0.9800
C1—F1	1.342 (3)	C8—H8A	0.9800
C1—C2	1.526 (4)	C8—H8B	0.9800
C2—O1	1.280 (3)	C8—H8C	0.9800
C2—C3	1.372 (4)	C9—O3	1.438 (4)
C3—C4	1.428 (4)	C9—H9A	0.9800
C3—H3	0.9500	C9—H9B	0.9800
C4—O2	1.254 (4)	C9—H9C	0.9800
C4—C5	1.536 (4)	O1—Zn1	2.054 (2)
C5—C8	1.523 (4)	O2—Zn1	2.040 (2)
C5—C6	1.536 (4)	O3—Zn1	2.161 (2)
C5—C7	1.547 (4)	O3—H3A	0.824 (18)
C6—H6A	0.9800	Zn1—O2^i^	2.040 (2)
C6—H6B	0.9800	Zn1—O1^i^	2.054 (2)
C6—H6C	0.9800	Zn1—O3^i^	2.161 (2)
C7—H7A	0.9800		
			
F2—C1—F3	106.6 (3)	C5—C8—H8A	109.5
F2—C1—F1	106.0 (3)	C5—C8—H8B	109.5
F3—C1—F1	106.3 (2)	H8A—C8—H8B	109.5
F2—C1—C2	114.7 (2)	C5—C8—H8C	109.5
F3—C1—C2	111.1 (3)	H8A—C8—H8C	109.5
F1—C1—C2	111.6 (2)	H8B—C8—H8C	109.5
O1—C2—C3	130.0 (3)	O3—C9—H9A	109.5
O1—C2—C1	112.4 (2)	O3—C9—H9B	109.5
C3—C2—C1	117.7 (3)	H9A—C9—H9B	109.5
C2—C3—C4	124.6 (3)	O3—C9—H9C	109.5
C2—C3—H3	117.7	H9A—C9—H9C	109.5
C4—C3—H3	117.7	H9B—C9—H9C	109.5
O2—C4—C3	123.5 (3)	C2—O1—Zn1	119.7 (2)
O2—C4—C5	116.9 (3)	C4—O2—Zn1	126.7 (2)
C3—C4—C5	119.6 (3)	C9—O3—Zn1	122.6 (2)
C8—C5—C4	110.1 (2)	C9—O3—H3A	112 (3)
C8—C5—C6	110.5 (2)	Zn1—O3—H3A	124 (3)
C4—C5—C6	111.6 (2)	O2^i^—Zn1—O2	180.0
C8—C5—C7	109.1 (3)	O2^i^—Zn1—O1^i^	89.64 (8)
C4—C5—C7	106.9 (2)	O2—Zn1—O1^i^	90.36 (8)
C6—C5—C7	108.5 (3)	O2^i^—Zn1—O1	90.36 (8)
C5—C6—H6A	109.5	O2—Zn1—O1	89.64 (8)
C5—C6—H6B	109.5	O1^i^—Zn1—O1	180.00 (11)
H6A—C6—H6B	109.5	O2^i^—Zn1—O3	89.95 (8)
C5—C6—H6C	109.5	O2—Zn1—O3	90.05 (8)
H6A—C6—H6C	109.5	O1^i^—Zn1—O3	89.97 (8)
H6B—C6—H6C	109.5	O1—Zn1—O3	90.03 (8)
C5—C7—H7A	109.5	O2^i^—Zn1—O3^i^	90.05 (8)
C5—C7—H7B	109.5	O2—Zn1—O3^i^	89.95 (8)
H7A—C7—H7B	109.5	O1^i^—Zn1—O3^i^	90.03 (8)
C5—C7—H7C	109.5	O1—Zn1—O3^i^	89.97 (9)
H7A—C7—H7C	109.5	O3—Zn1—O3^i^	179.999 (1)
H7B—C7—H7C	109.5		
			
F2—C1—C2—O1	177.3 (2)	C3—C2—O1—Zn1	20.4 (4)
F3—C1—C2—O1	56.2 (3)	C1—C2—O1—Zn1	−160.1 (2)
F1—C1—C2—O1	−62.2 (3)	C3—C4—O2—Zn1	−8.4 (4)
F2—C1—C2—C3	−3.2 (4)	C5—C4—O2—Zn1	173.6 (2)
F3—C1—C2—C3	−124.2 (3)	C4—O2—Zn1—O1^i^	−159.3 (2)
F1—C1—C2—C3	117.3 (3)	C4—O2—Zn1—O1	20.7 (2)
O1—C2—C3—C4	0.2 (5)	C4—O2—Zn1—O3	−69.4 (2)
C1—C2—C3—C4	−179.2 (3)	C4—O2—Zn1—O3^i^	110.6 (2)
C2—C3—C4—O2	−7.7 (5)	C2—O1—Zn1—O2^i^	155.1 (2)
C2—C3—C4—C5	170.3 (3)	C2—O1—Zn1—O2	−24.9 (2)
O2—C4—C5—C8	−17.8 (4)	C2—O1—Zn1—O3	65.1 (2)
C3—C4—C5—C8	164.0 (3)	C2—O1—Zn1—O3^i^	−114.9 (2)
O2—C4—C5—C6	−141.0 (3)	C9—O3—Zn1—O2^i^	−43.4 (2)
C3—C4—C5—C6	40.8 (4)	C9—O3—Zn1—O2	136.6 (2)
O2—C4—C5—C7	100.5 (3)	C9—O3—Zn1—O1^i^	−133.0 (2)
C3—C4—C5—C7	−77.6 (3)	C9—O3—Zn1—O1	47.0 (2)

Symmetry codes: (i) −*x*+2, −*y*+2, −*z*+1.

### Hydrogen-bond geometry (Å, °)

**Table:**

*D*—H···*A*	*D*—H	H···*A*	*D*···*A*	*D*—H···*A*
O3—H3A···O1^ii^	0.82 (2)	2.06 (2)	2.869 (3)	168 (4)

Symmetry codes: (ii) *x*−1, *y*, *z*.
